# Nasal and Nasopharyngeal Rosai-Dorfman Disease

**DOI:** 10.5334/jbsr.2896

**Published:** 2022-09-22

**Authors:** Kelly Di Dier, Marc Lemmerling, Geert De Vos

**Affiliations:** 1 AZ Sint Lucas, BE

**Keywords:** extranodal Rosai-Dorfman disease, nasal cavity, nasopharynx, computed tomography, magnetic resonance imaging

## Abstract

**Teaching Point:** Nasal and nasopharyngeal Rosai-Dorfman disease is a rare cause of nasal obstruction.

## Case History

**Figure 1:**
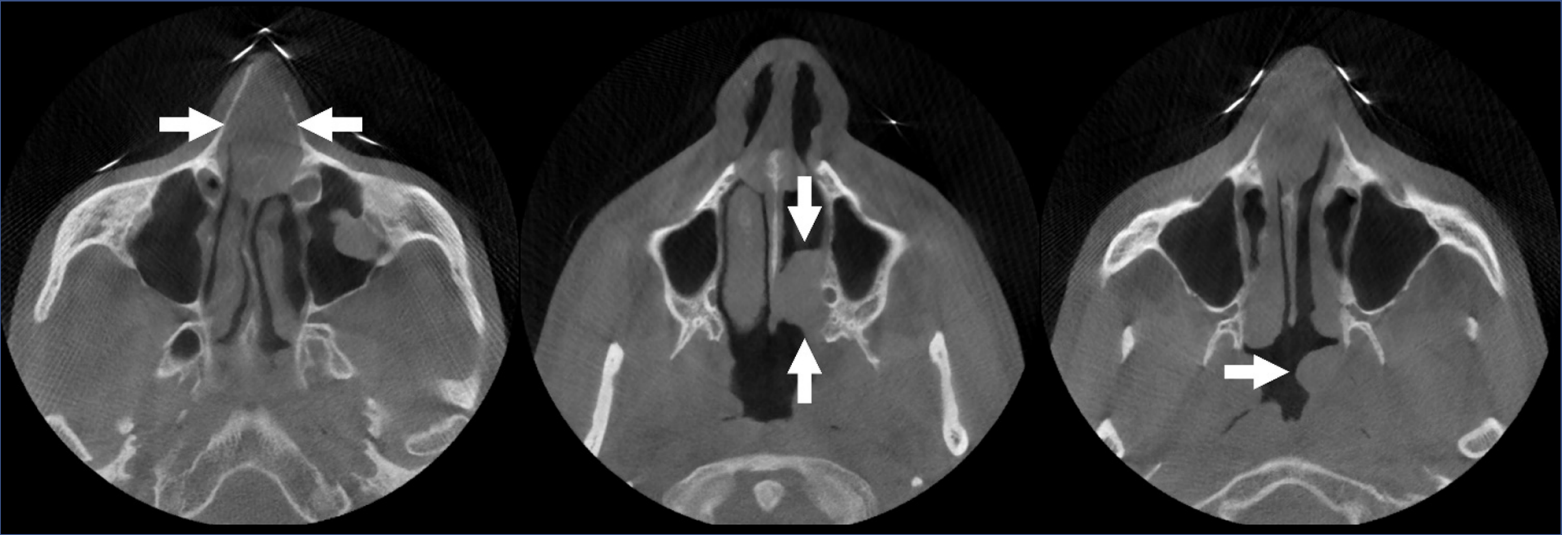


**Figure 2:**
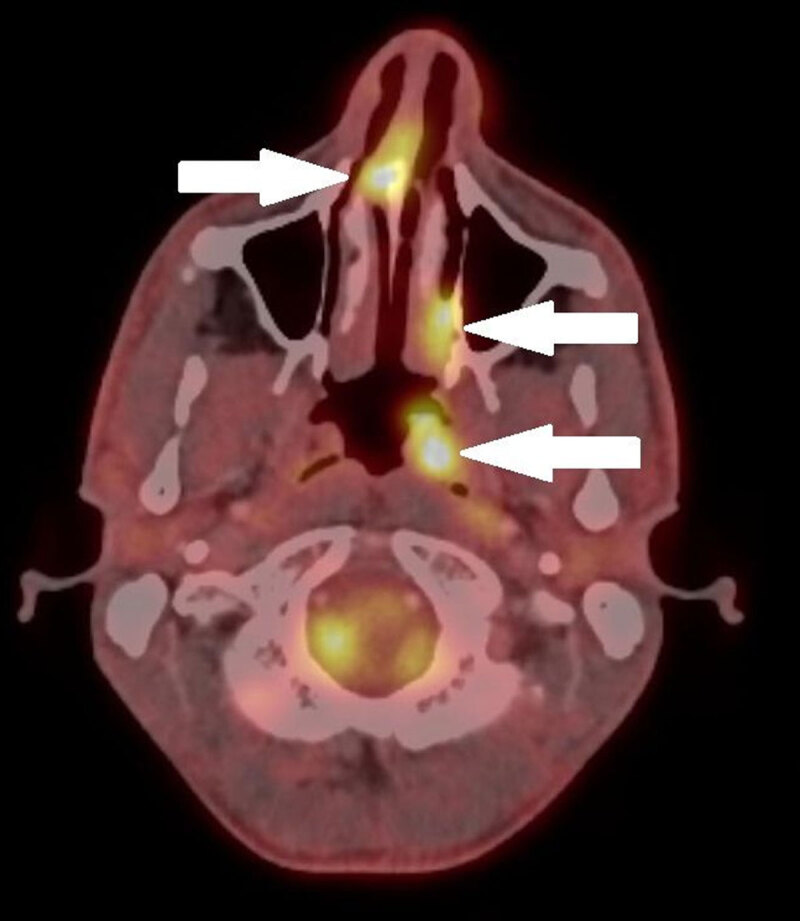


**Figure 3:**
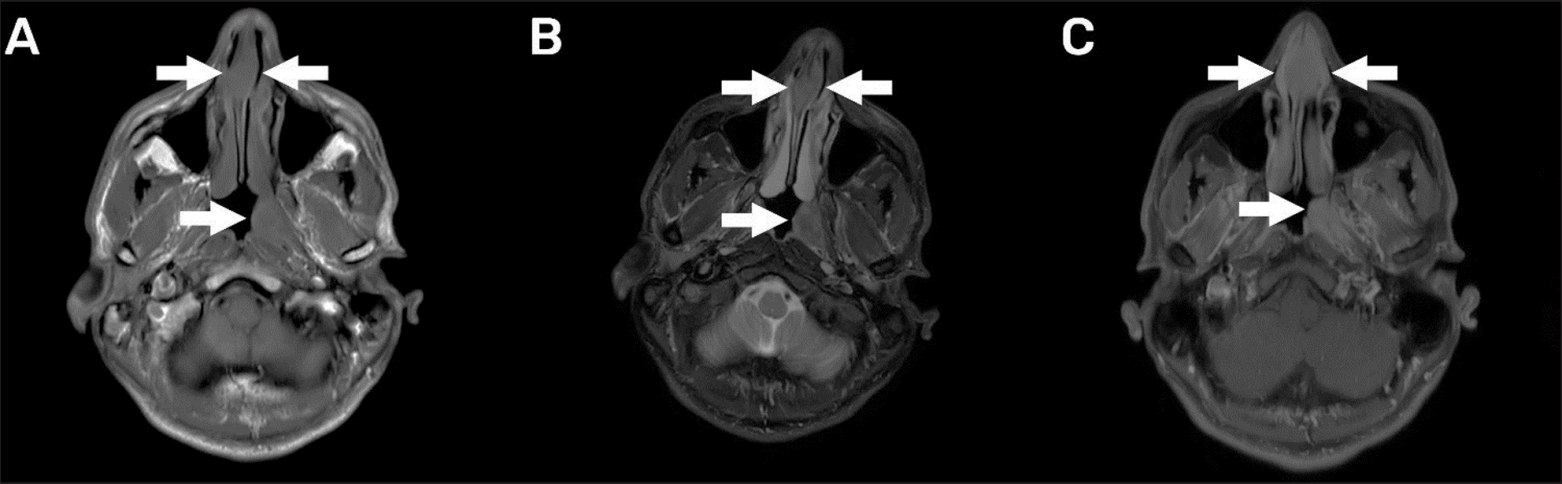


A 33-year-old male presented to the otolaryngologist with persistent complaints of nasal blockage. On clinical examination a nasal septum deviation was seen and was confirmed by subsequent cone-beam computed tomography (CT) of the sinuses, which also showed additional soft tissue masses in the anterior nasal septum, in the left-sided posterior nasal cavity, and at the level of the left torus tubarius (Figure 1). Suboptimal biopsy was performed with inconclusive histopathological result. Additional PET-CT showed high attenuation in the three lesions (Figure 2). Subsequent magnetic resonance imaging (MRI) of the sinuses and a renewed biopsy were executed. The lesions had a low signal intensity on the T1- and T2-weighted images (Figure 3A and 3B, respectively), and were iso-intense to slightly hypo-intense compared to grey matter. Moderate and homogeneous enhancement was present after intravenous contrast administration on the T1-weighted images with fat suppression (Figure 3C). Based on these findings, combined with a successful biopsy, extranodal Rosai-Dorfman disease was diagnosed.

## Comment

Sinus histiocytosis with massive lymphadenopathy, also known as Rosai-Dorfman disease, is a rare condition of idiopathic sinus histiocyte proliferation with incorporated phagocytosed lymphocytes. The etiology is not completely understood. A disturbed immunologic response against certain pathogens, more likely viruses, is presumed. This disease presents most frequently in the first two decades of life [1] and has a slight male predominance.

Rosai-Dorfman disease clinically presents with non-malignant and painless cervical lymphadenopathies or fever [1], which can be accompanied by night sweating, weight loss, and overall malaise. Extranodal manifestations are seen in up to one third of the patients and are more commonly found in the head and neck region, where they also tend to be multifocal. As in our patient, if present in the nasal region, complaints of nasal obstruction, rhinitis, and sinusitis can occur [1].

Non-contrast-enhanced CT will visualize a mass without further possibility to make a differentiation. The lesions have a high attenuation on FDG-PET CT, have an iso- to hypo-intense signal on T1- and T2-weighted MR images compared to grey matter, and homogeneously enhance after contrast administration. All of these imaging findings were present in this case.

Rosai-Dorfman disease is a benign pathology with an overall self-limiting course. Therefore, treatment is not necessary in most cases. Surgical intervention is executed for biopsy – with subsequent histological confirmation of the diagnosis – and rarely at local lesions to reduce concordant symptoms [1].

## References

[1] Hagemann M, Zbären P, Stauffer E, Caversaccio M. Nasal and paranasal sinus manifestation of Rosai-Dorfman disease. Rhinology. 2005; 43(3): 229–232. https://pubmed.ncbi.nlm.nih.gov/16218519 16218519

